# Establishment of bovine embryonic stem cells after knockdown of CDX2

**DOI:** 10.1038/srep28343

**Published:** 2016-06-20

**Authors:** Xia Wu, Miao Song, Xi Yang, Xin Liu, Kun Liu, Cuihua Jiao, Jinze Wang, Chunling Bai, Guanghua Su, Xuefei Liu, Guangpeng Li

**Affiliations:** 1 The Key Laboratory of National Education Ministry for Mammalian Reproductive Biology and Biotechnology, Inner Mongolia University, Hohhot 010070, China; 2 Department of Basic Medicine, Bao Tou Medical College, Bao Tou 014040, China; 3 Department of Pharmacy, Bao Tou Medical College, Bao Tou 014040, China; 4 College of Basic Medicine, Inner Mongolia Medical University, Hohhot 010110, China

## Abstract

Bovine embryonic stem cells (bESCs) have not been successfully established yet. One reason could be that CDX2, as the trophectoderm regulator, expresses in bovine inner cell mass (ICM), which probably becomes a technical barrier for maintaining the pluripotency of bESCs *in vitro*. We hypothesized that CDX2 knockdown (CDX2-KD) could remove such negative effort, which will be helpful for capturing complete and permanent capacity of pluripotency. Expression and localization of pluripotent genes were not affected in CDX2-KD blastocysts. The CDX2-KD bESCs grew into monolayers on feeder layer. Pluripotent genes expressed at an improved levels and lasted longer time in CDX2-KD bESCs, along with down-regulation of DNA methylation on promoters of both *OCT4* and *SOX2*. The cystic structure typical for trophoblast cells did not show during culturing CDX2-KD bESCs. CDX2-KD bESC-derived Embryoid bodies showed with compact morphology and with the improved levels of differentiations in three germ layers. CDX2-KD bESCs still carried the capacity of forming teratomas with three germ layers after long-term culture. In summary, *CDX2* in bovine ICM was inducer of trophoblast lineage with negative effect on maintenance of pluripotency of bESCs. Precise regulation CDX2 expression to switch on/off will be studied next for application on establishment of bESCs.

Embryonic stem cells (ESCs) have ability to differentiate into all cell lineages and germ cells. As the pluripotent stem cells, ESCs allow us to generate genetically modified animals useful for researches and applications, including biomedicine, agriculture and industry. They have been derived from the inner cell mass (ICM) of blastocysts from rodents and human^1^,^2^,^3^. However, bovine ESCs (bESCs) have not been successfully derived yet after numerous attempts based on the experience from rodents or human. Only the partially-featured ESCs in cattle were derived, showing with the incomplete capacities of chimeras formation and none of germ-line transmission^4^. All previous bESCs cannot be continuously passaged *in vitro* and their partial pluripotency gradually lost during culture^4^. There are several differences among mouse, human and cattle for early embryonic development. For example, embryonic implantation occurs in the uterus at embryonic day 5 (E5) for mouse and E7–9 for human. However, the blastocyst still floats in cow for 2–3 weeks before attached to the uterus of cows^5^.

The differences of developmental progress among several mammalian species are reflected by the cellular characteristics at blastocyst stage. Different from mouse, bovine E7′s trophectoderm (TE) cells showed with some characteristics of ESCs. For example, they expressed POU5F1 (OCT4)^6^, and had ability to contribute to the ICM when the dissociated TE cells aggregated with 8-cell embryos^7^. In addition, by analysis of deep sequencing, expression of TE genes *TEAD4*, *CDX2*, *YAP1* and *GATA3* was no difference between ICM and TE in cattle, showed that bovine ICM had different characteristics from mouse ICM that expression of these genes was restricted^8^,^9^.

CDX2 is key regulator for formation and functional maintenance of TE, which is necessary for the proliferation of TE cells in mouse, and played a pivotal role for establishment of TS cells *in vitro*^10^. CDX2 keeps expression in ICM of bovine but not mouse^9^. In mouse ICM and ESCs, expression of *CDX2* was repressed by the histone H3 Lys 9 (H3K9) methyltransferase (ESET) that interacted with *OCT4*^11^,^12^,^13^. To increase *CDX2* expression in ESCs negatively regulated *OCT4* and *NANOG* expression, induced them to differentiate into cells with trophoblast phenotype^10^,^14^, but CDX2 did not affect establishment of mouse ESC line though CDX2-deficient embryos failed to form blastocoel^15^,^16^. These findings suggested that CDX2 was not necessary to ICM formation, but induce the ES cell differentiation in mouse. Previous studies indicated that CDX2 were detectable in bovine ICM apart from TE^7^,^17^. Differ from CDX2-KD in mouse, the bovine CDX2-KD embryos could form blastocysts and development could even last up to 15 days after transfer into recipient cows^7^,^18^, but its function for development of bovine ICM and pluripotent maintenance of ESCs was unclear. Previously, bovine ICM cells that were isolated by immuno-surgery still showed trophoblast characteristics, such as cystic structure and cytoplasmic lipid inclusions during *in vitro* cultivation, suggested that the activation of CDX2 might induce trophoblast differentiation^19^. This finding suggested that CDX2 could be negative regulator for pluripotency of bESCs. Therefore, depletion of CDX2 in bovine embryos could recover pluripotent gene expressions from the repression state, thus benefit to establish bESCs.

In this study, bovine CDX2-KD embryos were generated after somatic nuclear transfer mediated *CDX2* knockdown. The bESCs were successfully derived from the ICM of CDX2-KD embryos. Our results revealed that CDX2-KD in bESCs significantly improved the maintenance of pluripotency. CDX2-KD bESCs colonies grew into monolayer during long-term cultivation. Compare to control cells, CDX2-KD bESCs showed the higher-level expression of pluripotent genes and the robust ability of *in vitro* and *in vivo* differentiations.

## Results

### Bovine blastocysts development was not affected after CDX2 knockdown

**Figure 1:**
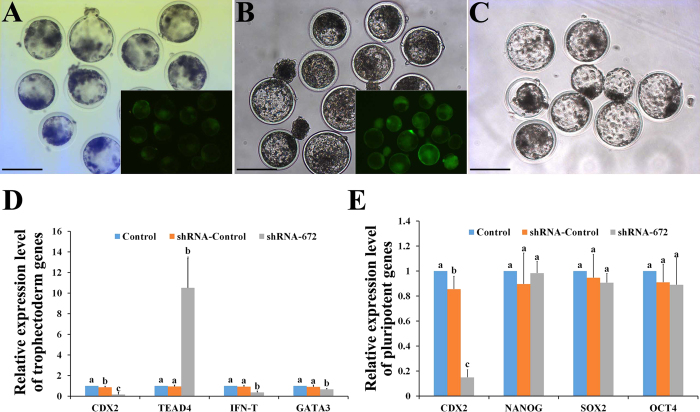
Generation of CDX2-KD blastocysts and quantitative analysis of TE and pluripotent genes. (**A**) Cloned blastocysts that expressed scrambled shRNA, GFP expression in these blastocysts was shown in inserted picture. Bar = 200 μm. (**B**) Cloned CDX2-KD blastocysts that expressed shRNA-672, GFP expression in these blastocysts was shown in inserted picture. Bar = 200 μm. (**C**) Cloned control blastocysts were generated by unmodified bEFs as donor. Bar = 200 μm. (**D**) The relative abundance of transcripts for *CDX2*, *TEAD4*, *IFNT* and *GATA3* in control, shRNA-Control and CDX2-KD blastocysts was determined. TE factors mRNA levels were normalized by β-actin mRNA level. Bars indicate mean ± SD. ^a/b/c^Values with different superscripts differ significantly (P < 0.05). (**E**) The relative abundance of transcripts for *OCT4*, *SOX2* and *NANOG* in control, shRNA-Control and CDX2-KD blastocysts was determined. Pluripotent factors mRNA levels were normalized by β-actin mRNA level. Bars indicate mean ± SD. ^a/b/c^Values with different superscripts differ significantly (P < 0.05).

**Figure 2:**
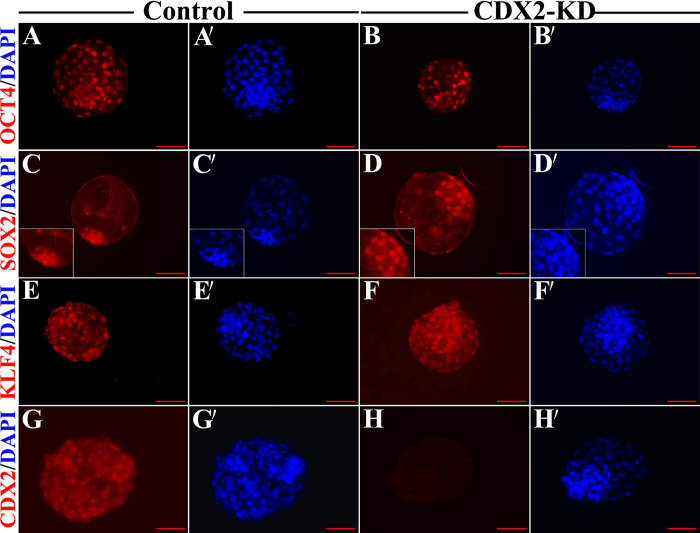
Expression and Localization of pluripotent associated factors in control and CDX2-KD blastocysts. (**A**, **A′** and **B**, **B′**) Immunofluorescent image of OCT4 at cell nucleus was fully positive in control and CDX2-KD blastocysts. (**C**, **C′** and **D**, **D′**) Immunofluorescent image of SOX2 at cell nucleus was positive in ICM and negative in TE in control and CDX2-KD blastocysts. ICM staining was shown in inserted picture. (**E**, **E′** and **F**, **F′**) Immunofluorescent image of KLF4 at cell nucleus was fully positive in control and CDX2-KD blastocysts. (**G**, **G′** and **H**, **H′**) Immunofluorescent image of CDX2 at cell nucleus was fully positive in control blastocyst, and was full negative in CDX2-KD blastocyst. Bar = 100 μm.

Space-temporal expressions for both mRNA and protein of CDX2 were first analyzed from oocytes to pre-implantation embryos, in order to design the strategy of *CDX2* gene knockdown and to evaluate the knockdown effects on the cultured bESCs afterward. Results indicated that *CDX2* mRNA was detectable at oocyte stage. After IVF manipulation, *CDX2* mRNA started to decrease gradually until 8-cell stage, and to increase afterward from morula to blastocyst stage (p < 0.05) (see Supplementary Fig. S1). On the other hand, CDX2 proteins were only detectable after blastocyst stage, localizing in the nuclei of both TE and ICM during the time from expanded to hatched blastocysts (see Supplementary Fig. S2). For obtaining the expected knockdown effect, optimal candidate of shRNA structure for knockdown of *CDX2* was first selected from a pool of our constructed DNA plasmids, including sequence fragments of shRNA-Control, shRNA488, shRNA621, shRNA672 and shRNA621 + 672, which were tested in the over-expressed *CDX2* bovine embryonic fibroblasts (O-C-bEFs) (see Supplementary Figs S3 and S4). The short hairpin RNA672 plasmid was selected for the highest efficiency known from comparison (see Supplementary Fig. S4). The SCNT embryos were derived from bEFs with the transfection of shRNA672 plasmid or shRNA-Control plasmid, as well as the control bEFs without transfection. The shRNA672 and shRNA-Control embryos expressed green fluorescent protein (GFP) that was driven by CMV promoter in pLL3.7 vector (Fig. 1A,B). Results showed that there was no difference for three kinds of SCNT embryos for the rates of cleavage, 8-cell and blastocyst as Supplementary Table S1. Remarkably, the CDX2-KD blastocysts formed normal blastocoel cavity and ICM, then hatched normally at Day 8 (Fig. 1A–C). Cell number and distribution in blastocysts showed that there were no differences among the three groups regarding cells of the ICM, total cell number, and the fraction of ICM cells over the total cell number, but TE cells were significantly decreased in CDX2-KD blastocysts compared to controls (Table 1, p < 0.05). The effect of knockdown in *CDX2* expression was proved in the CDX2-KD blastocysts (Fig. 1D,E, p < 0.01; and Fig. 2G,G′ and H,H′). As expected, the expression of TE genes *IFNT* and *GATA3* were down-regulated in CDX2-KD blastocysts in comparison with controls (Fig. 1D, p < 0.01). In the mean while, *TEAD4* as a number in TEAD transcription factor family was significantly up-regulated in CDX2-KD blastocysts in comparison with controls (Fig. 1D, p < 0.01). In addition, the core pluripotent regulators *OCT4*, *NANOG* and *SOX2* kept similar levels in both CDX2-KD and control blastocysts (Fig. 1E, p > 0.05), which also had similar cellular localizations (Fig. 2A,A′–D,D′). Further more, the KLF4 as pluripotent factors for maintaining ground-state of ESCs were also not affected for their gene expression levels and cellular localizations in CDX2-KD blastocysts. KLF4 signals normally located at cellular nuclei of ICM and TE (Fig. 2E,E′ and F,F′).

In summary, *CDX2* gene expressed during whole period of pre-implantation development and localized on TE and ICM of bovine blastocysts. CDX2 knockdown did not affected expression and localization of pluripotent genes, and development of ICM in bovine blastocysts.

### Establishment of bESCs from CDX2-KD blastocysts

**Figure 3:**
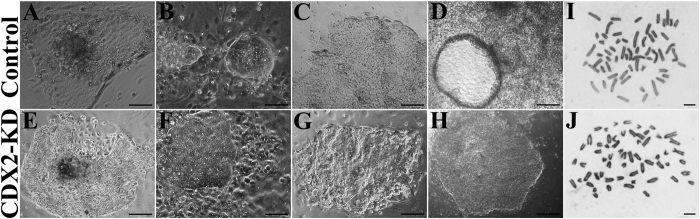
Morphology changes of bESCs during long-term cultivation and karyotype analysis. (**A,E**) ICM outgrowth appeared after 6 to 7 days of cultivation. Bar = 200 μm. (**B,F**) Domed colony similar to mouse ESCs at 3 passages was observed on MEFs feeder layer. Bar = 100 μm. (**C**) Morphology of control bESC colonies at passage 10 showing flat-shaped colonies similar to human ESCs. Bar = 200 μm. (**D**) Morphology of control bESC colonies at 15 passages formed cystic structure. Bar = 500 μm. (**G**) Morphology of CDX2-KD bESC colonies at 15 passages showing flat-shaped colonies similar to human ESCs. Bar = 200 μm. (**H**) CDX2-KD bESCs at 30 passages kept flat-shaped morphology without forming cystic structure. Bar = 500 μm. (**I**) Control bESCs at 17 passages showed normal karyotype. Bar = 5 μm. (**J**) CDX2-KD bESCs at 33 passages showed normal karyotype. Bar = 5 μm.

**Figure 4:**
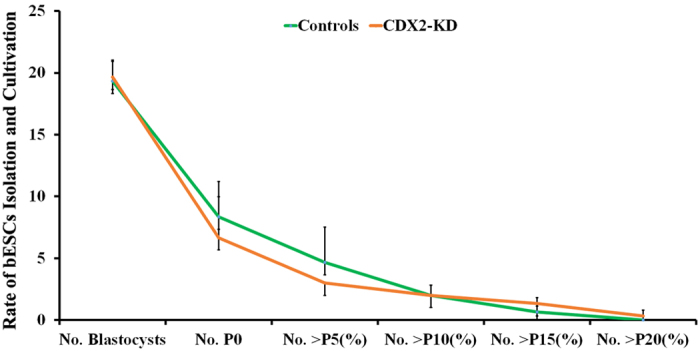
Efficiency of isolation and passage of bESCs derived from CDX2-KD and control blastocysts.

Next, ICM were mechanically isolated for outgrowth culture from 59 (19.67 ± 1.25) CDX2-KD early blastocysts at Day 6.5 to 7 and 58 (19.33 ± 1.70) controls at same stage. After transferring onto MEFs feeder layer, the ICM outgrowth around some polar trophoblasts was observed at day 6 to 7 (Fig. 3A,E), were mechanically separated from trophoblasts, and transferred onto new feeder layers. Subsequently, the colonies were then passaged by mechanical segmentation every 6 to 8 days. At early passages, the morphology of colonies was most similar with mouse ESCs for showing domed and compacted colonies with legible border (Fig. 3B,F). Subsequently, they gradually became to flatted monolayer, similar with human ESCs (Fig. 3C,G). Remarkably, some cystic structures emerged in the control bESCs, but not ever happened in CDX2-KD bESCs at the time of 30 passages (Fig. 3D,G,H). Results on both isolation and *in vitro* passage rates of bESCs suggested that CDX2-KD blastocysts had less efficiency to form preliminary expansions (Fig. 4 and see Supplementary Table S2). They were flimsy and did not survive well during early passages. After 10 passages, the passage rates of CDX2-KD bESCs were comparable with control bESCs (Fig. 4 and see Supplementary Table S2). After 15 passages, control bESCs mostly formed into cystic structures, only two bESCs could be passaged as cell lines similar as human ES cells in cultures, and none of them could be cultured beyond 20 passages as Supplementary Table S2. On the other hand, few CDX2-KD bESCs formed the cystic structures in comparison with control cells (Fig. 3H). A bESC line from CDX2-KD blastocysts was successfully cultured for over 30 passages. The CDX2-KD bESCs at 33 passages and control bESCs at 17 passages exhibited normal karyotype (Fig. 3I,J). In summary, bESCs were derived from bovine CDX2-KD blastocysts. During long-term cultivation, CDX2-KD bESCs kept consistent morphology similar as human ESCs, and stable proliferation. However, control cells showed trophoblast differentiation form cystic structures.

### Expression of pluripotent genes was well maintained in CDX2-KD bESCs during long-term cultivation

**Figure 5:**
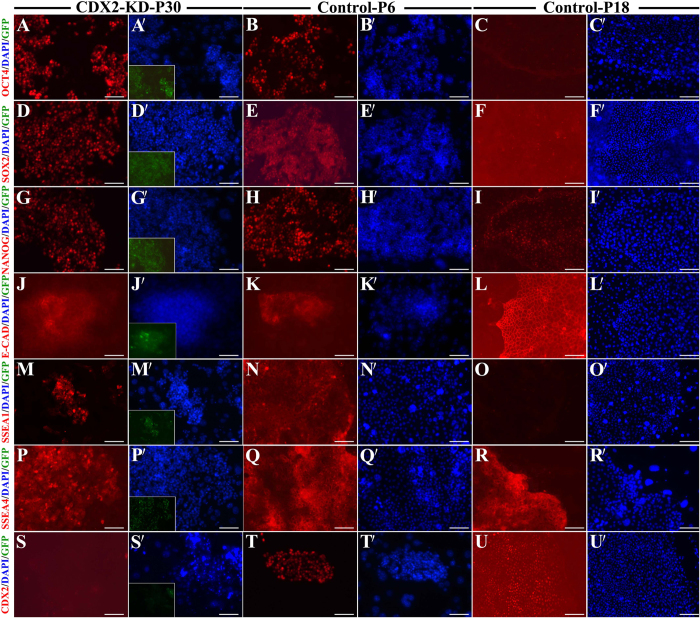
Expression of pluripotent markers and CDX2 in CDX2-KD and control bESCs. OCT4 (**A**, **A′**–**C**, **C′**), SOX2 (**D**, **D′**–**F**, **F′**), NANOG (**G**, **G′**–**I**, **I′**), E-CAD (**J**, **J′**–**L**, **L′**), SSEA1 (**M**, **M′**–**O**, **O′**), SSEA4 (**P**, **P′**–**R**, **R′**) and CDX2 (**S**, **S′**–**U**, **U′**). GFP expression in CDX2-KD bESCs was shown in inserted picture. Nuclear was stained by DAPI. Bar = 100 μm.

Expression of the pluripotent markers was analyzed in CDX2-KD bESCs at 6, 18 and 30 passages and control bESCs at 6, 18 passages (P6-, P18-bESCs) by immunocytochemistry. CDX2-KD bESCs robustly expressed pluripotent markers (OCT4, SOX2, NANOG, E-CADHERIN (E-CAD), SSEA1 and SSEA4) from 6 to 30 passages (Fig. 5AA′; D,D′; G,G′; J,J′; M,M′; P,P′, and see Supplementary Fig. S5), but CDX2 signals were negative (Fig. 5S,S′). Different from CDX2-KD bESCs, OCT4, SOX2 and SSEA1 signals were obviously detectable in P6-bESCs (Fig. 5B,B′; E,E′; N,N′), but lost in P18-bESCs (Fig. 5C,C′; F,F′; O,O′). NANOG, E-CADHERIN and SSEA4 abidingly expressed from P6- to P18-bESCs (Fig. 5H,H′; I,I′; K,K′; L,L′; Q,Q′; R,R′). In control bESCs, CDX2 staining was positive from 6 to 18 passages (Fig. 5T,T′; U,U′).

**Figure 6:**
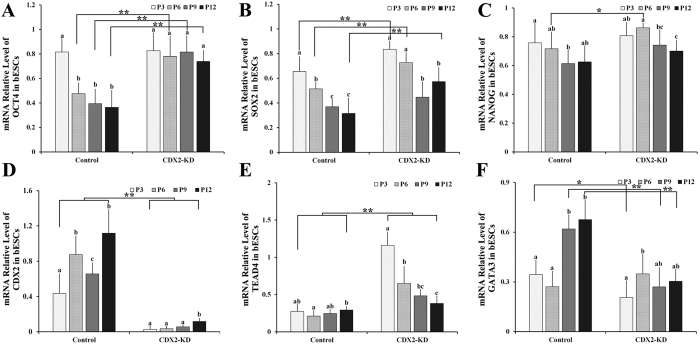
Expression of trophectoderm and pluripotent genes in CDX2-KD and control bESCs at different passages. These genes mRNA levels were measured by comparing to their abundance in blastocysts and normalized by β-actin mRNA level. Expression of *OCT4* (**A**), *SOX2* (**B**), *NANOG* (**C**), *CDX2* (**D**), *TEAD4* (**E**) and *GATA3* (**F**) in CDX2-KD and control bESCs at 3, 6, 9 and 12 passages. ^a/b/c^Values with different superscripts differ significantly (P < 0.05). *p < 0.05, **p < 0.01.

**Figure 7:**
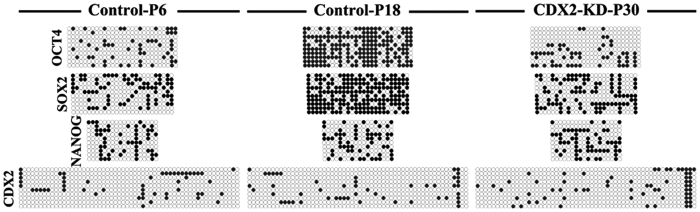
Bisulfite genomic sequencing of the promoter regions of *OCT4*, *SOX2*, *NANOG* and *CDX2* in CDX2-KD and control bESCs. Open circles indicate unmethylated CpG dinucleotides, while closed circles indicate methylated CpGs.

Quantitative analysis of TE and pluripotent genes in both bESCs at 3, 6, 9 and 12 passages showed that expression of *OCT4* in CDX2-KD bESCs kept at stable level from 3 to 12 passages (Fig. 6A, P > 0.05). However, its expression in control bESCs significantly decreased after 6 passages (Fig. 6A, P < 0.01). Compare with control bESCs, *OCT4* in CDX2-KD bESCs maintained a high-level expression (Fig. 6A, P < 0.01). Expression of *SOX2* in both bESCs was down-regulated from 3 to 12 passages (Fig. 6B, P < 0.01). Compared to the control bESCs, quantity of *SOX2* mRNA was significantly higher in CDX2-KD bESCs between different passages (Fig. 6B, P < 0.01). Expression of *NANOG* in both bESCs had a slight down-regulation during passages (Fig. 6C, P < 0.05). Apart from 6 passages, there was no significant difference between these two bESCs (Fig. 6C, P > 0.05). Further, analysis of DNA methylation revealed that promoters of *OCT4*, *SOX2* maintained low DNA methylation level in CDX2-KD bESCs, but were gradually methylated in control bESCs (Fig. 7). However, DNA methylation level of *NANOG* was no significant change in both bESCs (Fig. 7).

Expression of *CDX2* in CDX2-KD bESCs was significant silenced than that in control bESCs from 3 to 12 passages (Fig. 6D, P < 0.01). Its expression in control bESCs persistently increased from 3 to 12 passages (Fig. 6D, P < 0.01). At 12 passages of controls cells, expressing levels of *CDX2* were 1.12 times compared to it in the blastocysts. Low-level DNA methylation of *CDX2* promoter in both bESCs suggested that shRNA regulated repression of *CDX2* expression in CDX2-KD bESCs. *TEAD4* in CDX2-KD bESCs still maintained higher expression compared to that in the blastocysts, but kept down-regulation during passages (Fig. 6E, P < 0.01). However, low *TEAD4* expression was detected, and kept stably from 3 to 12 passages in control bESCs (Fig. 6E, P < 0.01), and its quantities were 0.27 times at 12 passages compared to that in the blastocysts. *GATA3* expression slightly increased in CDX2-KD bESCs from 6 to 12 passages, but was significantly up-regulated in control bESCs from 6 to 12 passages (Fig. 6F, P < 0.01). The expressing levels of *GATA3* at 9 and 12 passages were significantly higher in control bESCs than that in CDX2-KD bESCs (Fig. 6F, P < 0.01).

### Long-term cultured CDX2-KD bESCs maintained the capacity of three germ layer differentiation at more complete levels

**Figure 8:**
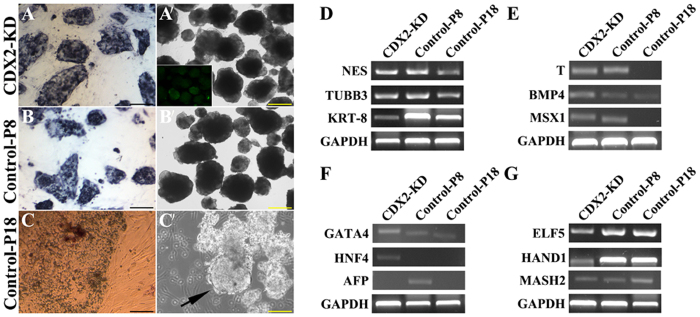
Expression of AKP and semi-quantitative analysis of EBs differentiation. Alkaline phosphatase staining of CDX2-KD bESCs (**A**), control bESCs at 8 passages (**B**) and 18 (**C**) passages. Bar = 200 μm. Compacted EBs derived from CDX2-KD bESCs at 34 passages (**A′**) and P8-bESCs (**B′**). GFP expression in CDX2-KD EBs was shown in inserted picture. Cystic embryoid bodies were derived from bESCs derived from P18-bESCs (**C′**). Bar = 500 μm. Express markers of three germ layers and trophoblast in these EBs. Ectodermal markers: *NES*, *TUBB3* and *KRT-8* (**D**). Mesodermal markers: *T*, *BMP4* and *MSX1* (**E**). Endodermal markers: *GATA4*, *HNF4* and *AFP* (**F**). Trophoblast markers: *ELF5*, *HAND1* and *MASH2* (**G**). GAPDH was used as a loading control.

Alkaline phosphatase (AKP) analysis also showed that CDX2-KD bESCs (34 passages) and control bESCs (8 passages) kept positive staining (Fig. 8A,B), but the signals were lost in control bESCs at 18 passages (Fig. 8C). Embryoid bodies were derived from these CDX2-KD bELSCs, and emerged two types of morphology. One was compacted EBs from P8-bESCs (P8-EBs) and CDX2-KD bESCs (CDX2-KD EBs) with the similar morphology to the mouse EBs (Fig. 8A′,B′). Another one from P18-bESCs (P18-EBs) was loose with blastocoels-like cavities (Fig. 8C′, Arrow). Semi-quantitative analysis of differentiation markers revealed that these EBs expressed ectoderm markers *NES*, *TUBB3* and *KRT-8*. High-level *NES* and *TUBB3* were detected in P8-EBs and CDX2-KD EBs. Compare to P8- and P18-EBs, *KRT-8* signals were weaker in CDX2-KD EBs (Fig. 8D). Mesoderm markers *BMP4*, *T* and *MSX1* expressed in P8-EBs and CDX2-KD EBs (Fig. 8E), but in P18-EBs, only *BMP4* signals were detectable (Fig. 8E). Endoderm markers, most intensive signals of *GATA4* were dectected in CDX2-KD EBs. *HNF4* was only detected in CDX2-KD EBs. *AFP* was detected in P8-EBs, but not in other groups (Fig. 8F). Presently, trophoblast markers *ELF5*, *HAND1* and *MASH2* were also investigated in these EBs. Low-level expression of these genes was detected in CDX2-KD EBs (Fig. 8G). However, control EBs expressed high-level *ELF5* and *HAND1*, while P18-EBs expressed highest-level *MASH2* (Fig. 8G).

**Figure 9:**
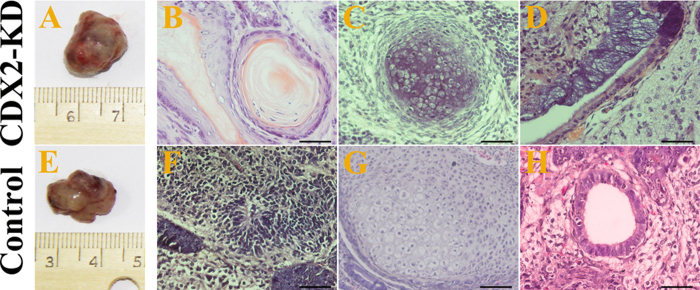
Teratomas formation and *in vivo* differentiation of bESCs. Teratoma from CDX2-KD bESCs was collected at day 74 after cell injection (**A**). Epidermis (**B**), cartilage (**C**) and columnar epithelium (**D**) were present in the teratoma. Bar = 50 μm. Teratoma from control bESCs at nascent passages was collected at day 88 after cell injection (**E**). Nerve tissue (**F**), cartilage (**G**) and columnar epithelium (**H**) were present in the teratoma. Bar = 50 μm.

Subsequently, we subcutaneously injected 5 × 10^6^ CDX2-KD bESCs at 37 passages, control bESCs at 5 to 8 and 15 to 18 passages into immuno-deficient mice. After 74 and 88 days, teratomas were collected from the recipient mice (Fig. 9A,E). However, no teratomas were derived from control bESCs at 15 to 18 passages. Histological examination revealed that these teratomas contained tissues from three germ layers, including epidermis (Fig. 9B), neural tissues (Fig. 9F), cartilage (Fig. 9C,G) and columnar epithelium (Fig. 9D,H). Altogether, long-term cultured CDX2-KD bESCs kept the ability of differentiation into all of the three germ layers *in vitro* and *in vivo*. For control bESCs, cellular pluripotency was lost, and revealed distinct trophoblast differentiation during long-term cultivation.

## Discussion

To obtain the real bESCs with fully defined characterizations of pluripotency is still an important and interesting study in the livestock ESC study field. Presently, name of bESC is given to the cells with partial characters of pluripotent stem cells, which were still short of capcity to generate chimeras and germ-line transmission. Limitation in understanding on molecular mechanism of bovine embryonic development could be one of the major theoretical hurdles. Many researchers have been trying on numerous strategies, including the imitation on the already established methods and culture systems for deriving and maintaining ESCs of both mouse and human. However, all of the already tested endeavors were not successful because the significant differences in embryonic development exist between bovine and mouse or human. Here, we showed that CDX2-KD in bovine blastocysts could be used to let our derived bESCs to capture features of pluripotency. Our results also suggested that sustaining expression of CDX2 could be one of the technical barriers for maintaining the derived bESCs. It is possible that bESCs with sustaining expression of CDX2 could automatically differentiate into trophoblast characteristics, which happen in bovine ICM, and systematically lose their pluripotency under the culture condition.

The mouse ESCs can be derived from CDX2-deficient embryos, although these embryos failing to maintain blastocoel cavities, hatch and implant, suggested that CDX2 was not required for development of ICM^15^,^16^. Similarly, loss of *CDX2* in cattle also had no impact on both the development of ICM and the expressions of pluripotent genes tested. Previous reports indicated that the expression and localization of pluripotent relative genes such as OCT4, SOX2 and NANOG were unchanged^7^,^18^. Our data further showed that CDX2-knockdown also did not affect the expression and localization of KLF4 gene. KLF4 expressed exclusively in human ICM, and have pivotal role in resetting ground-state of human ESCs^20^. Different from human, KLF4 expression was in both ICM and TE of bovine CDX2-KD and control blastocysts. Thus, our results suggested that there was no direct interaction between *KLF4* and *CDX2*. Together, knockdown of *CDX2* did not disturb pluripotency of ICM of CDX2-KD blastocysts. Differ from mouse, knockdown of *CDX2* in cattle revealed normal embryonic development, blastocyst formation and even develop for 15 days after transfer into recipient cows^7^,^18^, although expression of TE markers, *GATA3* and *IFNT* was significant down-regulation, respectively. However, *TEAD4* was significant up-regulation. In mouse, TEAD4 was essential for specification of the trophectoderm lineage when embryos developed to morula, activated expression of *CDX2* and *GATA3*, but was not necessary for functional TE formation^21^. GATA3, as another TE regulator was downstream of TEAD4 and in parallel to CDX2^22^. Mouse TEAD4-deficient embryos exhibited abnormal morphology, lacked blastocoel cavities and were disability to implantation *in vivo*^21^,^23^. However, no forthright proofs revealed that knockdown of *CDX2* up-regulated transcription of *TEAD4*. During development of bovine embryos, loss of *CDX2* may compensatorily up-regulate expression of *TEAD4*. Moreover, expression of *TEAD4* and residual *GATA3* may regulate bovine blastocysts formation when CDX2 was depleted.

Bovine CDX2 was expressed in TE and ICM with the unique cellular localizations, suggested that this should be specially considered when to establish bESCs. For testing our hypothesis, bovine CDX2-KD blastocysts and control blastocysts were used to generate ESCs, and the cells from two origins were compared during the process. Our results indicated that changes of colony morphology were first observed from compact dome-shaped to monolayer in both CDX2-KD and control bESCs during *in vitro* cultivation. After a relative long-term in culture, CDX2-KD bESCs stably maintained monolayer, but morphology of control bESCs started to form cystic structures, and accompanied up-regulation of *CDX2*. Cystic structures and cytoplasmic lipid inclusions are the typical feature of trophoblasts that usually emerge during culturing ESCs of domesticated ungulates, ever used the most stringent immuno-surgery method to isolate ICM^19^,^24^,^25^. In normal mouse ESCs, the trophoblast lineage was induced by ectopic expression of *TEAD4*, *GATA3* and *CDX2*. However, *CDX2* is essential for establishment of trophoblast lineages in ESCs because over expression of *TEAD4* and *GATA3* in mouse CDX2-deficient ESCs failed to induce TS cell lines^22^,^26^. Here, our findings in cattle agree with these previous reports. Although low-level expression of *TEAD4* and *GATA3* were detectable in CDX2-KD bESCs, the trophoblast lineage did not induce, which revealed that CDX2 is also a key inducer for bovine trophoblast differentiation.

Previous studies revealed that pluripotent genes had low-level expression and some were even silenced in previous bESCs, suggesting a non-power pluripotent network^27^,^28^. In our study, mRNA expression levels of *OCT4* and *SOX2* were maintained at the higher levels in CDX2-KD bESCs when compared with controls, and their gene loci were not silenced by DNA methylation. *CDX2* was repressed by *OCT4*, and the ectopic expression of *CDX2* negatively regulated *OCT4* expression in both mouse ESCs and bovine trophectoderm CT-1 cells^10^,^29^. Therefore, the up-regulated *CDX2* in our control bESCs induced the trophoblast fate for these cells along with the repressed expression of *OCT4* and *SOX2*, and broke the core network that was composed of *OCT4*, *SOX2* and *NANOG* during *in vitro* cultivation.

Formations of embryoid bodies and teratomas are the essential evidences for proving the pluripotency of ESCs. For mouse and human ESCs, the round and compact embryoid bodies are usually formed during the process that cells undertake the automatic differentiation *in vitro*^30^,^31^. However, compact or cystic EBs formed from previous bESCs, which show no difference in capacity of differentiation^19^,^32^,^33^. In our study, compact EBs were derived from CDX2-KD bESCs at 34 passages and P8-bESCs. Cystic EBs were derived from P18-bESCs. However, these two kinds of EBs showed different ability of differentiation, the compact EBs were more robustly expressed markers of three germ layers than the cystic one, while the latter maintained high-level expression of trophoblast markers, tended to differentiate into trophoblasts lineage. In addition, for control bESCs, the ability of teratomas formation was lost during *in vitro* cultivation, showed that the CDX2-KD bESCs stably maintained pluripotency, while the control bESCs lost their pluripotency, and differentiated into trophoblast fate during long-term cultivation.

In summary, CDX2-KD in bovine embryos did not affect bovine pre-implantation development and expressions of pluripotent genes, which were derived CDX2-KD bESCs successfully. In comparison with previous type of bESCs, CDX2-KD bESCs kept their morphology more stably, and kept expression of pluripotent genes at higher level, as well as the more robust ability of differentiation *in vitro* and *in vivo*, because CDX2-KD resulted in inhibition on the fate to become trophoblast during long-term cultivation. On the contrary, previous type of bESCs as controls emerged trophoblast characteristics and lost their pluripotency because expression of *CDX2* was up-regulation during *in vitro* cultivation. Altogether, CDX2 as a barrier to pluripotent maintenance of bESCs was removed after CDX2-KD, which can be beneficial for establishment of bovine embryonic stem cells, and it is necessary to develop novel culturing system for establishing bESC lines with some small molecular compounds that inhibit the cells from differentiating into trophoblast lineage.

## Materials and Methods

Unless otherwise mentioned, all reagents used were purchased from Life Technologies Company (USA). All studies adhered to procedures were in accordance with the National Research Council Guide for the Care and Use of Laboratory Animals and were approved by the Institutional Animal Care and Use Committee at Inner Mongolia University.

### Lentiviral production, infection and cell culture

The lentiviral expressing and packaging vectors, pSin-EF2-OCT4-Pur, pLL3.7, psPAX2 and pMD2.G, were purchased from Addgene (USA). *CDX2* gene was cloned from small intestine of bovine fetus, and replaced *OCT4* in pSin-EF2-OCT4-Pur, named pSin-EF2-bCDX2-Pur. Short hairpin RNAs (shRNA) were designed using the BLOCK-iT RNAi Designer (developed by Life Technologies Company), which is freely available at http://rnaidesigner.lifetechnologies.com/rnaiexpress/design.do. Three functional shRNAs that impacted on 488 bp-506 bp, 621 bp-640 bp and 672 bp-691 bp of *CDX2* gene were synthesized, and Cloning into pLL3.7 backbone, named pLL3.7-shRNA488, pLL3.7-shRNA621, pLL3.7-shRNA672. A vector that included scrambled shRNA was also constructed, named pLL3.7-shRNA-Control. The shRNA sequences were provided in Supplemental Table S3.

Expressing vectors together with packaging vectors at ratio of 3:2:1 (pSin-EF2-bCDX2-Pur or pLL3.7: psPAX2: pMD2.G) were transfected into 293FT cells using Lipofectamine 2000 following the instructions of the manufacturer. Sixteen hours after transfection, the medium was replaced. Two days later, virus-containing supernatant was collected, centrifuged at 1500 × g for 10 min at 4 °C and removed cellular debris. The lentivirus was concentrated by Lenti-X Concentrator (Clontech), and re-suspended in proper fibroblast medium afterward. Virus supernatant supplemented with 8 μg/ml polybrene was used to infect bEFs at 3 passages. After two round infections in two days, virus-containing medium was replaced with fresh medium. For establishing CDX2-overexpression cell lines, the medium was added into 0.8 μg/ml puromycin to select transfected cells at day one after infection.

### Production of IVF and SCNT embryos

The IVF and SCNT embryos were produced as described previously^34^. Bovine oocytes sourced from a local slaughterhouse were matured and fertilized *in vitro*. After IVF, cumulus cells were removed from presumptive zygotes by mouth pipette, and cultured in SOFaa supplemented with 8 mg/ml bovine serum albumin (fatty acid free, fraction V, Sigma), 1% (v/v) non-essential amino acids (100×), 2% (v/v) essential amino acids (50×), 1 mM L-glutamine, 0.4 mM sodium pyruvate and 50 μg/ml gentamycin in 40 μl droplets overlaid with mineral oil at 38.5 °C, 5% CO_2_ in a humidified atmosphere for 48 h. The cleaved embryos were transferred to 40 μl droplets of SOFaa with 4% FBS under humidified atmosphere of 5% CO_2_ in air at 38.5 °C. For analyzing dynamic changes of expression and localization of CDX2, oocytes with the first polar body were collected. Two-cell, 8-cell, morula, early blastocyst, expanded blastocyst and hatched blastocyst were collected at 24, 48, 120, 156, 168 and 204 h after co-incubating oocytes with spermatozoa, respectively.

After oocyte maturation, cumulus cells were removed by vortex in 0.1% hyaluronidase (Sigma) for 4 min. Oocytes with first polar body were enucleated under an inverted microscope. Single bEF was transferred to the perivitelline space of enucleated recipient cytoplasts. The couplets were sandwiched with a pair of electrodes, and two electric DC pulses of 1.8 kV/cm for 20 μs were delivered by a Voltain cell fusion system (Cryologic). At IVM 25–26 h, fused embryos were chemical activation by treatment with 5 μM ionomycin for 5 min, then 10 μg/ml cycloheximide (CHX) for 5 h in SOF medium. The activated embryos were cultured in SOFaa medium for 48 h. Cleaved reconstructed embryos were transferred into 40 μl drops of SOFaa containing 4% FBS until they developed to blastocysts stage.

### Differential staining of blastocysts

The quality of blastocysts was assessed by differential staining of ICM and TE cells according to previous protocol^35^, with minor modifications. Blastocysts at day 7 were incubated in 500 μl of BSA-free, HEPES-buffered TCM-199 with 1% Triton X-100 and 100 μg/ml PI for 30 s, and then fixed overnight in 500 μl 100% ethanol with 25 μg/ml of Hoechst 33258 at 4 °C. The blastocysts were mounted on a glass microscope slide in a drop of 50% glycerol, gently flattened with a coverslip and observed using epifluorescence microscopy.

### Isolation and culture of bovine ES cells

SCNT blastocysts with a well-developed ICM on Day 6.5 or Day 7 were used for isolating ICM as described previously^7^. Zonae pellucidae of these blastocysts were removed with 1% pronase (Sigma), and were manually split into two portions. Part of containing the ICM was pressed onto mouse embryonic fibroblast (MEF) feeder layer inactivated with mitomycin C (10 μg/ml; Sigma), and allowed to form primary embryo outgrowths. The ICM was cultured in a single well of 4-well or 24-well plate (Nunc) containing 1 ml medium consisting of basic ES medium (KnockOut-Dulbecco modified Eagle medium supplemented with 2 mM Glutamine, 1% MEM nonessential amino acids, 20 ng/ml human recombinant basic fibroblasts growth factor, 20 ng/ml recombinant human LIF (Millipore), and 0.1 mM β-mercaptoethanol) supplemented with 15% ES cell qualified FBS (Hyclone) under 37 °C, 5% CO_2_. After 6 to 7 days of culture, compact ICM outgrowth was removed by a glass pipette and transferred onto new feeder layers with basic ES medium supplemented with 10% FBS and 10% KnockOut-Serum Replacement (KSR). The medium was changed every two days. About one week, the colonies were broken into small pieces by mouth pipette, gently, and then transferred onto new feeder layer with basic ES medium supplemented with 20% KSR.

### Karyotype analysis

CDX2-KD bESCs at 33 passages and control bESCs at 17 passages were arrested in metaphase by incubating them in culture medium containing 0.2 μg/mL colcemid (KaryoMax). The cells were harvested by Accutase treatment, washed in PBS, and then resuspended in 0.075 M KCl for 30 min at 37 °C. After centrifugation at 1,000 rpm for 5 min, the cells were fixed twice in cold fixative (1:3 glacial acetic acid/methanol). Chromosome spreads were prepared by dropping cells suspension onto cold slides. After drying, the slides were stained with Giemsa solution.

### Alkaline phosphatase staining and immunofluorescence analysis

Alkaline phosphatase staining was performed with BCIP/NBT Alkaline Phosphatase Colour Development Kit (Beyotime) according to manufacturer’s instructions. For immunostaining, embryos or cells were fixed with 4% paraformaldehyde for 30 min and then permeabilized with 1% Triton X-100 for 30 min followed by blocking with 2% BSA (Sigma). Cells were incubated in primary antibody overnight at 4 °C and secondary antibody at 37 °C for 1 h. Primary antibodies were used that were anti-OCT-3/4 (Santa Cruz), anti-NANOG (Abcam), anti-SOX2 (Cell signaling), anti-SSEA1 (Santa Cruz), anti-SSEA4 (Santa Cruz), anti-E-CADHERIN (BD Bioscience), anti-KLF4 (Stemgent), anti-CDX2 (Biogenex).

### Methylation analysis

CDX2-KD bESCs (30 passages) and control bESCs (6 and 18 passages) were used to analysize promoter methylation of *OCT4*, *SOX2*, *NANOG* and *CDX2*. DNA treatment and methylation specific PCR was executed by EpiXplore Methyl Detection Kit (TaKaRa) and EpiTaq HS (for bisulfite-treated DNA) Kit (TaKaRa) according to manufacturer’s instructions. The PCR primers to amplify methylation regions of these four genes as described previously as Supplementary Table S4 36. The PCR products were ligated into pBackZero-T-Vector (TaKaRa) for methylation sequencing. More than 10 clones per gene were sequenced and analyzed for each sample.

### Quantitative and semi-quantitative RT-PCR

Total RNA was isolated from cells using MiniBEST Universal RNA Extraction Kit (TaKaRa). RNAs from 15–20 blastocysts were prepared by Arcturus Picopure RNA Isolation Kit according to manufacturer’s instructions. RNA quality and quantity were examined using a NanoDrop 2000C Spectrophotometer (Thermo Scientific). Before reverse transcription (RT), concentration of total RNA was adjusted to 100 ng/μl. RT reaction solution was prepared which contained 500 ng RNA, RNase Free H_2_O and PrimeScript RT Master Mix (TaKaRa) according to manufacturer’s instructions. RT reaction was performed on MasterCycler Nexus (Eppendorf). Quantitative analysis of cDNA was performed by Applied Biosystems 7500 Real-Time PCR System. PCR reactions were performed by initially denaturing cDNA at 95 °C for 30 s, which was followed by 40 cycles of denaturing at 95 °C for 5 s, annealing for 34 s at 60 °C. For semi-quantitative RT-PCR, equal amount of cDNA was added into the reaction, and PCR products were ran electrophoresis on a 1.5% agarose gel. The primer sequences for quantitative and semi-quantitative RT-PCR are provided in Supplemental Table S5.

### *In Vitro* and *In Vivo* differentiation assay

For preparing embryoid bodies, a hanging drop method was used according to previously studies with some modifications^37^,^38^. Before preparing EBs, bESCs were cultured at high concentration in 100 mm dishes. After rinsing cultured bESCs with PBS 3 to 4 times, add Accutase, a cell detachment solution to coat the bottom of the dish (2 ml/100 mm dish). Incubate cells at 37 °C incubator until cells detached bottom, added into 5 ml differentiation medium consisting of DMEM/F12 supplemented with 15% FBS (Hyclone), 2 mM Glutamine, 1% MEM nonessential amino acids, gently collected the cell clumps into 10 ml conical tube, kept the tube for half hour under 38.5 °C, 5% CO_2_, let the clumps converge to pyramis of the tube, carefully removed supernatant and added the differentiation medium, re-suspended and did not disturb cell clumps. Adjusted the stem cell suspension to a concentration of 30 to 50 clumps per 50 μl (50 μl/drop). Add 10 ml of PBS into petri dish. Lifted lid, made rows of 50 μl drops on the up-turned inner surface of the lid, carefully inverted it. Carefully place the dish in the incubator for 3 days. After three days, carefully turn over the dish cover, gently removed the medium including dead cells, did not disturb growing EBs and added fresh differentiation medium, place the dishes into the incubator undisturbed for 3 days, repeat the medium changes according status of the EBs. Embryoid body was collected one-by-one by mouth pipette. Semi-quantitative RT-PCR was performed to detect expressing difference of mark genes of three germ layers in these EBs.

For producing teratomas, 5 × 10^6^ CDX2-KD bESCs at 37 passages, control bESCs at 5–8 passages and 15–18 passages were injected subcutaneously into groin and oxter of 6- to 8-week-old NOD-SCID mice. After 10 to 12 weeks, teratomas were isolated and fixed in 4% paraformaldehyde, embedded in paraffin. Histological sections were stained with hematoxylin/eosin.

### Statistical analysis

Statistical analysis for results of bovine cloned embryonic development, isolation and cultivation of bESCs, and qPCR were performed on Microsoft Excel to acquire standard error of the mean (SEM). Statistically significant differences between groups were identified using t-test.

## Additional Information

**How to cite this article**: Wu, X. *et al*. Establishment of bovine embryonic stem cells after knockdown of CDX2. *Sci. Rep.* **6**, 28343; doi: 10.1038/srep28343 (2016).

## Supplementary Material

Supplementary Information

## Figures and Tables

**Table 1:** Cell allocation of bovine somatic nuclear transfer blastocysts on Day 7.

	**ICM**	**TE**	**Total**	**ICM/Total (%)**
CDX2-KD	30.63 ± 6.02^a^	64.5 ± 6.70^a^	95.13 ± 10.02^a^	33.51 ± 8.32^a^
shRNA-Control	29.11 ± 8.22^a^	68.26 ± 8.60^ab^	97.37 ± 13.29^a^	29.60 ± 5.70^a^
Control	28.25 ± 5.21^a^	73.63 ± 13.00^b^	101.88 ± 14.40^a^	28.44 ± 4.43^a^
