# Supplementary material for: Establishment of bovine embryonic stem cells after knockdown of CDX2
Source: Sci Rep. 2016 Jun 20;6:28343. doi: 10.1038/srep28343 (PMC4913270; doi:10.1038/srep28343)
Supplement: Supplementary Information [file srep28343-s1.pdf]

Title: Establishment of bovine embryonic stem cells after knockdown of CDX2

Authors: Xia Wu[1,†,*], Miao Song[2,3,†], Xi Yang[3,†], Xin Liu[1,†], Kun Liu[1], Cuihua Jiao[1], Jinze Wang[1], Chunling Bai[1], Guanghua Su[1], Xuefei Liu[1], Guangpeng Li[1,*]

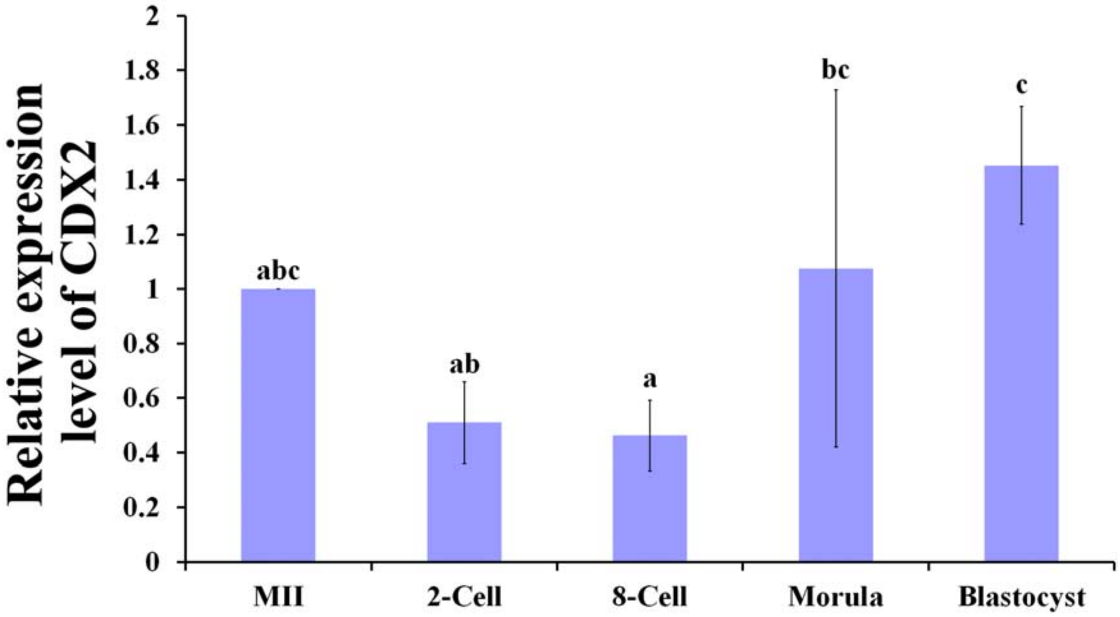

Supplementary Figure S1 Expression of *CDX2* in bovine MII oocytes and pre-implantation embryos. Quantitative RT-PCR analysis of *CDX2* expression in bovine oocytes (22h IVM) and embryos (156h post-IVF). In each set of experiments, *CDX2* mRNA levels were normalized by β-actin mRNA level. Bars indicate mean±SD. [a/b/c]Values with different superscripts differ significantly ($p < 0.05$).

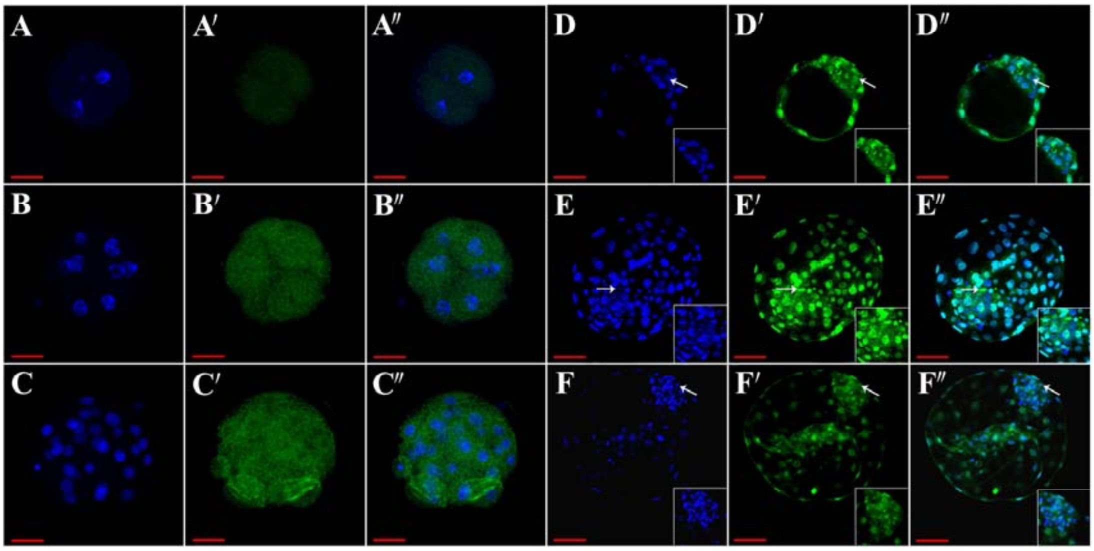

Supplementary Figure S2 Localization of CDX2 in bovine pre-implantation embryos. The staining patterns of IVF embryos in 2-cell (A, A', A''), 8-cell (B, B', B''), morula (C, C', C''), early blastocyst (D, D', D''), expanded blastocyst (E, E', E'') and hatched blastocyst (F, F', F''). ICM staining was shown in inserted picture. Nuclei are stained with DAPI. Bar = 100μm.

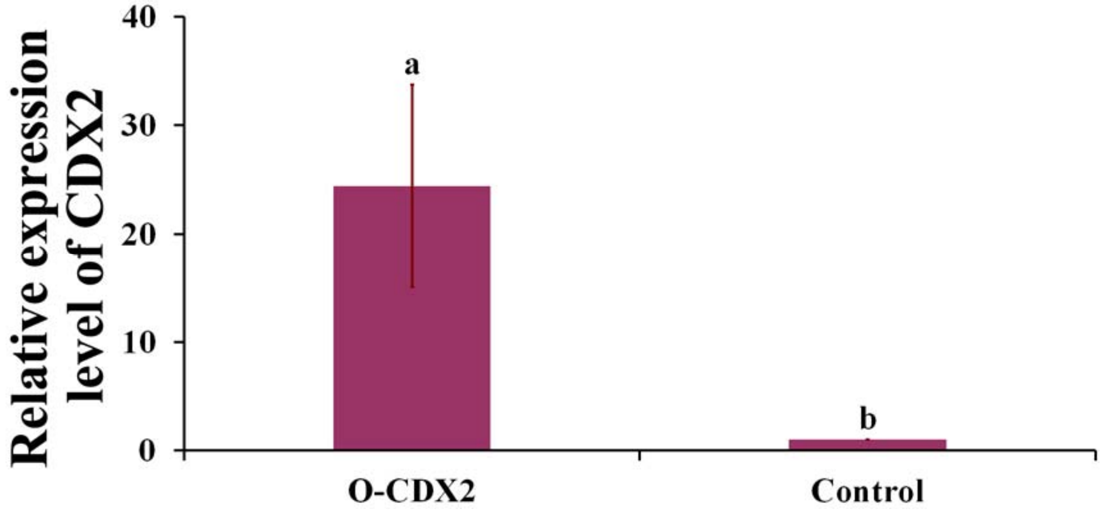

Supplementary Figure S3 The qRT-PCR analysis of *CDX2* expression in bEFs that was infected with containing *CDX2* over-expression plasmid for 48h. The relative expression of *CDX2* was quantitative analysis after post-infection 48hr. *CDX2* mRNA levels were normalized by β-actin mRNA level. Bars indicate mean±SD. [a/b]Values with different superscripts differ significantly ($P<0.01$).

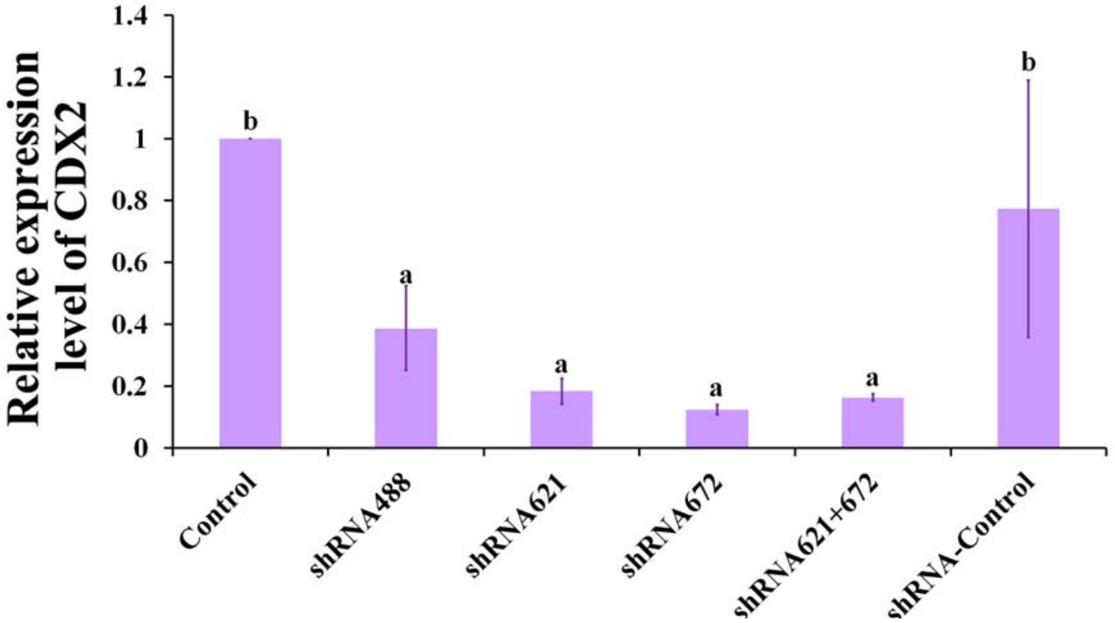

Supplementary Figure S4 The qRT-PCR analysis of *CDX2* expression in its over-expression bEFs after infecting lentivirus containing *CDX2* knockdown plasmids. The cells were detected at day one after infection. *CDX2* mRNA levels were normalized by β-actin mRNA level. Bars indicate mean±SD. [a/b]Values with different superscripts differ significantly (P<0.01).

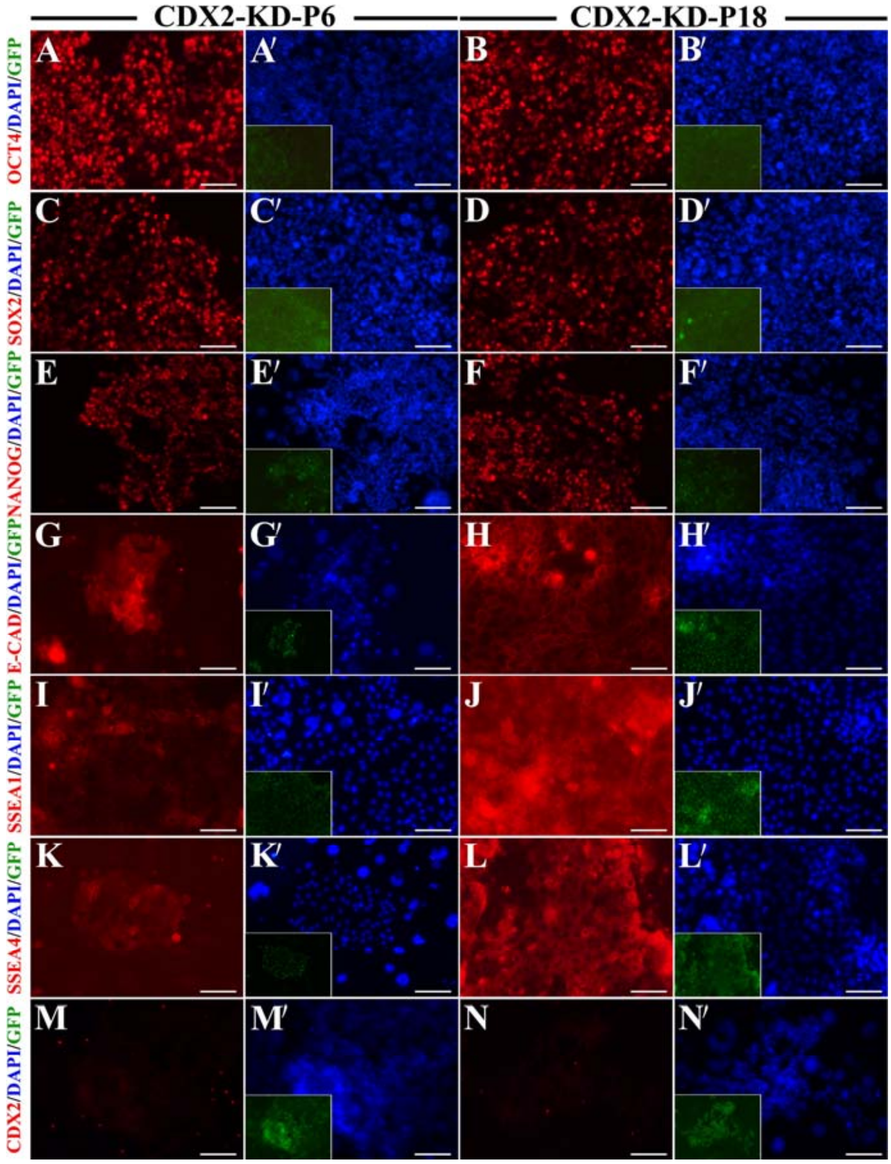

Supplementary Figure S5 Expression of pluripotent markers and CDX2 in CDX2-KD bESCs at 6 and 18 passages. OCT4 (A, A', B, B'), SOX2 (C, C', D, D'), NANOG (E, E', F, F'), E-CAD (G, G', H, H'), SSEA1 (I, I', J, J'), SSEA4 (K, K', L, L') and CDX2 (M, M', N, N'). GFP expression in CDX2-KD bESCs was shown in inserted picture. Nuclear was stained by DAPI. Bar = 100μm.

	No. reconstructed embryos	No. Cleavage (%)	8-cell (%)	Blastocyst (%)
Control	271	209(77.12±2.67)a	124(45.76±8.04)a	79(29.15±3.36)a
shRNA-Control	242	193(79.75±1.99)a	105(43.39±3.71)a	73(30.17±4.26)a
shRNA-672	266	216(81.2±2.82)a	127(47.74±1.1)a	70(26.32±1.83)a

Supplementary Table S1 Development of cloned embryos derived from CDX2-KD bEFs

	No. Blastocysts	No. P0(%)	No. >P5(%)	No. >P10(%)	No. >P15(%)	No. >P20(%)
CDX2-KD	59	20(34.2±16.63)a	9(15.33±1.01)a	6(10.5±4.87)a	4(6.8±2.29)a	1(1.67±22.36)a
Control	58	25(42.12±11.41)a	14(29.33±21.13)a	6(10.07±3.43)a	2(3.27±2.31)a	0(0)a

Supplementary Table S2 Isolation and cultivation of bESCs from CDX2-KD and control blastocysts

Fuctional Sites in *CDX2* (bp)	Forward Sequence of shRNA	Reverse complementary Sequence of shRNA
**448-506**	AACGGAACCTGTGCGAGTGGATTTCAAGAGAATCCACTCGCACAGGTTCCTTTTTTC	TCGAGAAAAAAGGAACCTGTGCGAGTGGATTCTCTTGAAATCCACTCGCACAGGTTCCGTT
**621-640**	AACGCAGTCGCTATATCACCATCTTCAAGAGAGATGGTGATATAGCGACTGCTTTTTTC	TCGAGAAAAAAGCAGTCGCTATATCACCATCTCTCTTGAAGATGGTGATATAGCGACTGCGTT
**672-691**	AACGCTCTCAGAGAGGCAGGTTATTCAAGAGATAACCTGCCTCTCTGAGAGCTTTTTTC	TCGAGAAAAAAGCTCTCAGAGAGGCAGGTTATCTCTTGAATAACCTGCCTCTCTGAGAGCGTT
**Scramble**	AACGAGGTGGTTCGATGACTCATTCAAGAGATGAGTCATCGAACCACCTCTTTTTTC	TCGAGAAAAAAGAGGTGGTTCGATGACTCATCTCTTGAATGAGTCATCGAACCACCTCGTT

Supplementary Table S3 shRNA for silencing *CDX2* in this study

Gene	Forward Primer	Reverse Primer
**OCT4**	TGGGTCGGGAGGGTTAGAGT	CAACAACTCACTCGCCTCCTC
**SOX2**	GCGTTTTTTTTTTTATTTTAGTAGT	ACTTTCCCCCTTTTACAAACA
**NANOG**	AGGGATTGAAGGTTATTTGTTTT	TATCCAAACATCCAAAAATTAAAA
**CDX2**	TGGAGGGGCGTAGGGTTTA	ACTCCTACGCCGACGAACAA

Supplementary Table S4 Primers used in this study for DNA Methylation Analyses

Gene	Forward Primer s (5'→3')	Reverse Primers (5'→3')	Amplicon Size in bp	Gene ID
Real-time PCR Primers				
OCT4	CAAATTAGCCACATCGCC	AGCCTCAAAATCCTCACG	126	NM_174580.2
SOX2	TCAGATGCAGCCCATGCAC	GGTGCCCTGCTGAGAATAGGAC	121	NM_001105463.1
NANOG	ATCTGCTGACACCCTCGACAC	GGGTCTGCGAGAACAGTTCTAA	193	NM_001025344.1
CDX2	AGTGAAAACCAGGACGAAAGA	CTCTGAGAGCCCCAGCGT	142	XM_871005.3
TEAD4	AAGTTCTGGGCAGACCTCAA	GTGCTTCAGCTTGTGGATGA	249	XM_605145.3
GATA3	ATGAAACCGAAACCCGATG	TTCACAGCACTAGAGAGACC	185	NM_001076804.1
IFN-T	GATCCTTCTGGAGCTGGYTG	GCCCGAATGAACAGACTCYC	100	NM_001168275.1
β-ACTIN	CTGTTAGCTGCGTTACACCCTT	TGCTGTCACCTTCACCGTTC	165	AY141970.1
Semi-Quantitiy RCR Primers				
NESTIN	CGCATGAATGGGGGCGTGGT	TTTGGAAGGGCCGCTGAGCC	565	JN180938
CYTOKERATIN-8	GTCTCCAGGCTGAGATCGAG	TCCAGCAGCTTCCTGTAGGT	241	NM_001033610.1
β3-TUBLIN	CATCCAGAGCAAGAACAGCAG	GATTCCTCCTCATCGTCTTCG	336	NM_001077127
T	TGATCACCAGCCACTGCTTC	CAGCATATCTTTGTGATCGC	149	NM_001192985
MSX1	GGACTCCTCAAACTGCCAGA	TACTGCTTCTGGCGGAACTT	224	NM_174798
BMP4	GGTAACCGAATGCTGATGGT	CTGGGATGTTCTCCAGATGT	385	NM_001045877.1
GATA4	AAGTCCTTCTCCCCTGTTTCC	GAGCAGAATCACCAAGAGCAG	383	XM_005209826.2
HNF4	ACATCCCAGCCTTCTGTGAG	TCGTCAATCTGCAGCTCTTG	238	AF250028
AFP	TCTTCCCCATGTTCTTCAGG	TTTCACGGCAAATTTCTTCC	205	NM_001034262
ELF5	GCTTGAAAACAAGTGGCATC	TCTTCCTTTGTCCCCACATC	207	NM_001024569.1
HAND1	ACATCGCCTACCTGATGGAC	TAACTCCAGCGCCCAGACT	215	NM_001075761.1
MASH2	CGACCGGAGTCCCCAGA	GCTCAACTTCTTGCTGGCAC	189	DQ381723.1
GAPDH	TCAAGAAGGTGGTGAAGCAG	CCCAGCATCGAAGGTAGAAG	122	GU324291.1

Supplementary Table S5 Semi-Quantitiy RCR and Real-time PCR primers
